# Developmental delay in a *Streptomyces venezuelae glgE* null mutant is associated with the accumulation of *α*-maltose 1-phosphate

**DOI:** 10.1099/mic.0.000296

**Published:** 2016-07

**Authors:** Farzana Miah, Maureen J. Bibb, J. Elaine Barclay, Kim C. Findlay, Stephen Bornemann

**Affiliations:** ^1^​ Department of Biological Chemistry, John Innes Centre, Norwich Research Park, Norwich NR4 7UH, UK; ^2^​ Department of Molecular Microbiology, John Innes Centre, Norwich Research Park, Norwich NR4 7UH, UK; ^3^​ Department of Cell and Developmental Biology, John Innes Centre, Norwich Research Park, Norwich NR4 7UH, UK

**Keywords:** *Streptomyces*, glucan, polysaccharide, GlgE, glycogen, sporulation

## Abstract

The GlgE pathway is thought to be responsible for the conversion of trehalose into a glycogen-like *α*-glucan polymer in bacteria. Trehalose is first converted to maltose, which is phosphorylated by maltose kinase Pep2 to give *α*-maltose 1-phosphate. This is the donor substrate of the maltosyl transferase GlgE that is known to extend *α*-1,4-linked maltooligosaccharides, which are thought to be branched with *α*-1,6 linkages. The genome of *Streptomyces venezuelae* contains all the genes coding for the GlgE pathway enzymes but none of those of related pathways, including *glgC* and *glgA* of the glycogen pathway. This provides an opportunity to study the GlgE pathway in isolation. The genes of the GlgE pathway were upregulated at the onset of sporulation, consistent with the known timing of *α*-glucan deposition. A constructed Δ*glgE* null mutant strain was viable but showed a delayed developmental phenotype when grown on maltose, giving less cell mass and delayed sporulation. Pre-spore cells and spores of the mutant were frequently double the length of those of the wild-type, implying impaired cross-wall formation, and spores showed reduced tolerance to stress. The mutant accumulated *α*-maltose 1-phosphate and maltose but no *α*-glucan. Therefore, the GlgE pathway is necessary and sufficient for polymer biosynthesis. Growth of the Δ*glgE* mutant on galactose and that of a Δ*pep2* mutant on maltose were analysed. In both cases, neither accumulation of *α*-maltose 1-phosphate/*α*-glucan nor a developmental delay was observed. Thus, high levels of *α*-maltose 1-phosphate are responsible for the developmental phenotype of the Δ*glgE* mutant, rather than the lack of *α*-glucan.

## Introduction

Glycogen is a type of *α*-glucan polymer that is widespread among bacteria, yeasts and mammals (Preiss, 2009). Its primary role is thought to be as a store of carbon and energy such that it is often synthesized during times of nitrogen limitation. Glycogen is composed of linear chains of *α*-1,4-linked glucose rings that are connected through *α*-1,6-linked branch points giving a tree-like structure with about 10 % branching. The classical glycogen biosynthetic pathway starts with the pyrophosphorylase GlgC that converts glucose 1-phosphate and ATP to ADP-glucose and pyrophosphate. The *α*-1,4-links are generated by glycogen synthase GlgA, using ADP-glucose as the donor in bacteria. The *α*-1,6-linked branches are introduced by the branching enzyme GlgB, which transfers a portion of the non-reducing end of the polymer onto a hydroxyl group at the six position of an internal glucose ring along a linear part of the polymer.

**Fig. 1.**
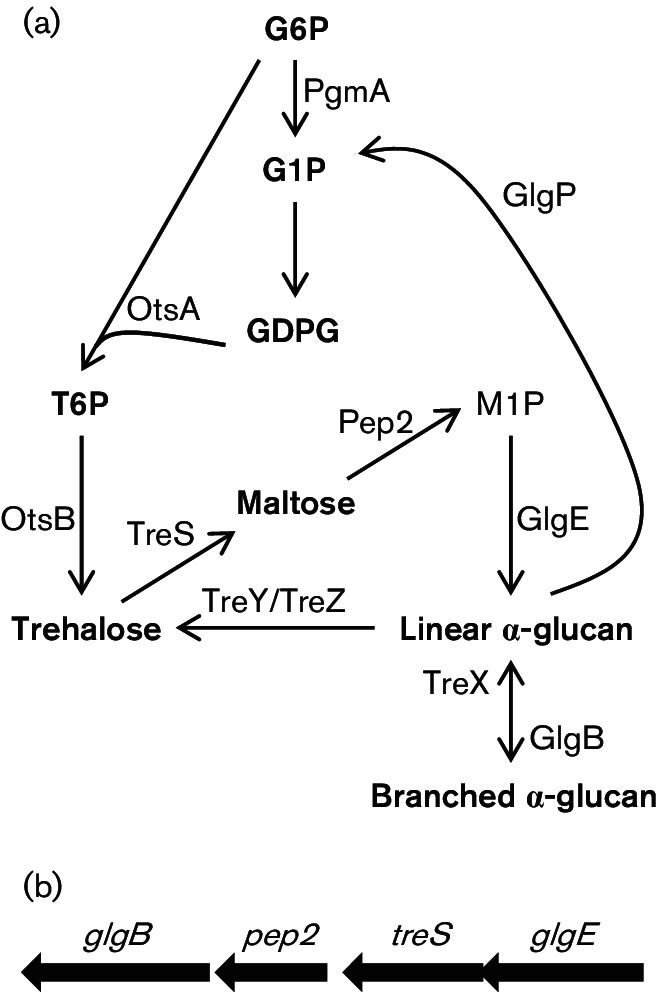
(a) The proposed metabolic pathways associated with the production of *α*-glucan in *Streptomyces venezuelae*. G6P, glucose 6-phosphate; G1P, glucose 1-phosphate; GDPG, GDP-glucose; T6P, trehalose 6-phosphate; M1P, *α*-maltose 1-phosphate. (b) The genes associated with the GlgE pathway in the context of the* S. venezuelae* genome.

The GlgE pathway (Fig. 1a) was recently discovered and is thought to provide an alternative route to *α*-glucans in bacteria (Bornemann, 2016). In this case, the *α*-1,*α*-1 linked non-reducing disaccharide trehalose is first isomerized to *α*-1,4-linked *α*-maltose (Pan* et al.*, 2004). The *α*-maltose is converted to *α*-maltose 1-phosphate by maltose kinase Pep2 (Jarling* et al.*, 2004; Niehues* et al.*, 2003). The *α*-maltose 1-phosphate is used as a donor by GlgE to generate *α*-1,4 linkages (Elbein* et al.*, 2010; Kalscheuer* et al.*, 2010). Although GlgE has been demonstrated to extend malto-oligosaccharides somewhat *in vitro*, there have been no reports of *in vivo* or *in vitro* experimental evidence supporting the notion that GlgE is capable of generating very large polymers. The branching enzyme GlgB, which is common to both pathways, is expected to introduce the branches into linear molecules of sufficient length (Garg *et al.*, 2007). The genes that encode the enzymes of the GlgE pathway are present in 14 % of sequenced bacterial genomes (Chandra* et al.*, 2011). Such bacteria include Gram-positive and Gram-negative species, particularly those with large genomes and complex lifestyles, suggesting that the GlgE pathway is fairly widespread (Chandra* et al.*, 2011). This compares with a 32 % occurrence for the classical glycogen pathway genes.

The significance of the GlgE pathway is exemplified in its likely association with the virulence of *Mycobacterium tuberculosis*. This human pathogen not only contains glycogen, but also coats itself in an *α*-glucan, which is the major component of the outer capsule (Lemassu & Daffé, 1994; Schwebach* et al.*, 2002). The *α*-glucan has been shown to be associated with virulence (Sambou* et al.*, 2008), most probably by interacting with immune receptors such as DC-SIGN (Cywes* et al.*, 1997; Gagliardi* et al.*, 2007; Geurtsen* et al.*, 2009). *M. tuberculosis* possesses the genes for not only the GlgE pathway but also the classical GlgA pathway. Although it has yet to be established how the two pathways contribute to the synthesis of the intra- and extra-cellular *α*-glucans in this organism, the GlgE pathway has been genetically validated as a drug target (Kalscheuer & Jacobs, 2010; Kalscheuer* et al.*, 2010). The mode of killing is unusual. Blocking either GlgE or GlgB leads to the accumulation of cytosolic *α*-maltose 1-phosphate to a toxic level that kills the bacterial cells. By contrast, the ability to block TreS or Pep2 shows that the loss of the entire GlgE pathway and its *α*-glucan is tolerated. This may be because of redundancy, seemingly not between the GlgE and classical pathways, but between the GlgE and the Rv3032 pathway (Kalscheuer* et al.*, 2010), which is responsible for the biosynthesis of methylglucose lipopolysaccharides. These comprise an *α*-glucan backbone of up to about 20 glucose rings decorated with methyl and acyl groups, and are thought to be involved in chaperoning the biosynthesis of long-chain fatty acids (Kaur* et al.*, 2009; Stadthagen* et al.*, 2007). Synthetic lethality between the GlgE and the Rv3032 pathways, but not between the GlgE and GlgA pathways, therefore implies that methylglucose lipopolysaccharides are essential in *M. tuberculosis* rather than *α*-glucan.

Fellow actinomycetes of the *Streptomyces* genus are also known to produce *α*-glucan and trehalose (Braña* et al.*, 1982). These soil-dwelling mycelial organisms have a complex developmental life cycle in which substrate hyphae give rise to reproductive sporulating aerial hyphae (Bush* et al.*, 2015; Flardh & Buttner, 2009). The amounts of *α*-glucan and trehalose can be substantial. For example, *Streptomyces antibioticus* can accumulate *α*-glucan up to 20 % dry cell weight and trehalose up to 12 % dry weight in aerial hyphae and spores (Braña* et al.*, 1986). Interestingly, glycogen accumulation in the model organism *Streptomyces coelicolor* occurs transiently in two distinct phases. Phase I deposition occurs in the substrate mycelium where aerial branches emerge, while phase II deposition occurs in the tips of aerial hyphae that are undergoing sporulation. Ultimately, trehalose accumulates in spores as a protectant from various abiotic stresses (Martin* et al.*, 1986) and provides energy and carbon during germination, at which time it is hydrolysed to glucose by trehalase (Hey & Elbein, 1968; McBride & Ensign, 1990).

The GlgE pathway is present in many actinomycetes such as *S. coelicolor* (Chandra* et al.*, 2011). Interestingly, this organism has two copies of the four GlgE pathway genes cluster, with each cluster being transcribed as a poly-cistronic mRNA (Plaskitt & Chater, 1995; Schneider* et al.*, 2000). The two clusters are developmentally regulated such that one is associated with phase I deposition and the other with phase II (Bruton* et al.*, 1995; Plaskitt & Chater, 1995). Consistent with this, phase II genes are reliant on WhiG (Yeo & Chater, 2005), a sigma factor that initiates sporulation septation. It is likely that the degradation of *α*-glucan in the pre-spore stage is by the debranching enzyme TreX, an isomerase TreY and a hydrolase TreZ to produce trehalose destined for the spores. Consistent with this, *treZ* is regulated by a sporulation-specific transcription factor (Bush* et al.*, 2013). Additional levels of post-translational allosteric and phospho-regulation of the GlgE pathway have also been identified (Asención Diez* et al.*, 2015; Leiba* et al.*, 2013).

It is not known whether the production of *α*-glucan is essential for *S. coelicolor* or whether blocking GlgE leads to cell death associated with the accumulation of *α*-maltose 1-phosphate. Indeed, there is no evidence directly linking the GlgE pathway with the production of *α*-glucan* in vivo*, with no *α*-glucan-deficient mutant of any species that possesses the GlgE pathway having been described to date. While it is possible to use reverse genetics in *S. coelicolor* to help address these questions, this would be challenging, because this organism contains not only two copies of the genes for the GlgE pathways but also the genes for the classical GlgA pathway (Chandra* et al.*, 2011). We therefore examined the genomes of other actinomycete organisms (Chandra* et al.*, 2011) and found that *S. venezuelae* had only one copy of the genes encoding the enzymes of the GlgE pathway (Fig. 1b) and neither *glgC* nor *glgA* of the classical pathway (Fig. 1a). It may therefore not be a coincidence that the transient deposition of only phase II *α*-glucan has been observed with this organism (Ranade & Vining, 1993). *S. venezuelae* also has two sets of genes associated with the formation of the precursor for the GlgE pathway, trehalose. It has TreX, TreY and TreZ, allowing the recycling of *α*-glucan, as well as OtsA and OtsB, which are responsible for the conversion of glucose 6-phosphate and GDP-glucose into trehalose 6-phosphate followed by dephosphorylation to give trehalose (Elbein, 1967). The relative simplicity of the *α*-glucan metabolic pathways in *S. venezuelae* provided an opportunity to establish a link between the GlgE pathway and the production of *α*-glucan* in vivo,* and to determine the effects of blocking the production of *α*-glucan and of accumulating *α*-maltose 1-phosphate on the viability of cells and the developmental lifecycle of this organism.

## Methods

### Bacterial strains, plasmids, oligonucleotides and growth conditions.

The strains, plasmids and oligonucleotides used in this study are detailed in Table S1, available in the online Supplementary Material. Plasmids and cosmids were propagated using *Escherichia coli* DH5*α*. Disruption cosmids were created using *E. coli* BW25113 (Datsenko & Wanner, 2000), containing a *λ* RED plasmid, pIJ790. Cosmids were conjugated from the *E. coli* strain ET12567 containing pUZ8002 (Gust* et al.*, 2003, 2004; Paget* et al.*, 1999). *S. venezuelae* strains were normally cultured at 28 °C in MYM-TAP (Kieser* et al.*, 2000) made with 50 % tap water and supplemented with 0.4 ml of trace element solution (Kieser* et al.*, 2000) per litre. The minimal medium contained (per litre) 1 g (NH_4_)_2_SO_4_, 0.5 g K_2_HPO_4_, 0.2 g MgSO_4_.7H_2_O, 0.01 g FeSO_4_.7H_2_O, pH 7.0, 10 g Iberian agar, 0.4 ml of trace element solution and 5 g of either maltose or galactose (Kieser* et al.*, 2000). Conjugation between *E. coli* and *S. venezuelae* was carried out as described previously (Kieser* et al.*, 2000), except that spores were not heat-shocked prior to mating and plates were incubated at room temperature overnight before overlaying with the selective antibiotics. Spores of *S. venezuelae* strains were gently harvested from MYM-TAP plates using 3 ml of 20 % (v/v) glycerol and sterile cotton pads (Bush* et al.*, 2013), unless stated otherwise.

### Construction and complementation of *S. venezuelae* null mutants.

Null mutants of *S. venezuelae* in *glgE* (gene locus synonyms SVEN_5097 and SMD07732), *pep2* (SVEN_5095, SMD07729) and *treS* (SVEN_5096, SMD07731) were generated using the Redirect PCR targeting method (Gust* et al.*, 2003) in which the coding regions were replaced with a single apramycin resistance (*apr*) cassette. We made use of a cosmid library that covers >98 % of the *S. venezuelae* genome (M. J. Bibb and M. J. Buttner, unpublished) as described fully at http://strepdb.streptomyces.org.uk/. The cosmid SV-3-D04 was introduced into *E. coli* BW25113 containing pIJ790, and the relevant gene was replaced with the *apr–oriT* cassette amplified from pIJ773 using the appropriate so-called disfor and disrev primer pairs (Table S1). The resulting disrupted cosmids were confirmed by restriction digestion and introduced into *S. venezuelae* by conjugation. Null mutants generated by double cross-over were identified by resistance to apramycin and sensitivity to kanamycin. Their chromosomal structures were confirmed using PCR analysis with the appropriate flanking confor and conrev primer pairs. Additional confirmation was provided by Southern hybridization using, as a probe, the cosmid partially digested with *Xcm*I or *Bam*HI for the *glgE* mutant, and *Pst*I for the *pep2* mutant. For complementation, the appropriate gene was amplified with the appropriate comfor and comrev primers to give a fragment comprising the coding sequence and ~300 bp upstream, which included its endogenous promoter. The fragment was cloned into the *Eco*RV restriction site of pMS82 (Gregory* et al.*, 2003). The resulting plasmids were introduced into the appropriate mutants by conjugation.

### Metabolite analysis using NMR spectroscopy.

For each strain, a 75 µl aliquot containing a set number of colony forming units (typically 10^6^) of a standardized spore stock was evenly distributed on a sterile cellophane disc covering the surface of solid medium in a Petri dish. The inoculated plate was incubated at 30 °C for an allotted period and the cells were harvested by scraping the cellophane, freeze-dried, and powdered using a micro pestle to fragment hyphae. The cells were then re-suspended in water (25 mg in 800 µl), boiled for 8 min to denature enzymes and disrupt cells, cooled on ice and sonicated on ice for 10 cycles of 30 s on and 30 s off with a Sonics Vibra-Cell VCX 500 Ultrasonic Processor set at 40 % amplitude to complete cell lysis. Light microscopy showed that cells were fully lysed. The cell debris was pelleted by centrifugation at 30 000* g* for 30 min at 4 °C. Typically, 540 µl of the cell-free extract was mixed with 60 µl of D_2_O and 3 µl of sodium 3-(trimethylsilyl)propionate-2,2,3,3-d_4_. ^1^H NMR spectra were recorded using a Bruker Avance III 400 spectrometer using standard pulse sequences and a probe temperature of 25 °C at 400 MHz with solvent-suppression. Chemical shifts are expressed in parts per million (p.p.m.) relative to sodium 3-(trimethylsilyl)propionate-2,2,3,3-d_4_ (0 p.p.m.). Spectra were analysed using Topspin 3.0 (Bruker) and resonances were integrated manually. The concentrations of trehalose (anomeric doublet at ~5.19 p.p.m.) and maltose (reducing end *α*-anomeric doublet at ~5.24 p.p.m. and *α*-1,4 link anomeric doublet at ~5.41 p.p.m.) were determined from their NMR resonances (Miah* et al.*, 2013), using sodium 3-(trimethylsilyl)propionate-2,2,3,3-d_4_ as an internal standard. Note that the chemical shifts reported previously (Miah* et al.*, 2013) were quoted relative to H_2_O at 4.70 p.p.m. rather than an internal standard, so are shifted by about 0.1 p.p.m. It was possible to distinguish between maltose and glucose, which share identical reducing end *α*-anomeric resonances, by only the former having an associated resonance for an *α*-1,4 linkage together with different chemical shifts associated with their *β*-anomeric doublet resonances (~4.67 and ~4.66 p.p.m., respectively) (Miah* et al.*, 2013). It was possible to determine the concentration of *α*-maltose 1-phosphate because its resonances (phosphorylated anomeric doublet of doublets at ~5.46 p.p.m. and *α*-1,4 link anomeric doublet at ~5.43 p.p.m.) are distinct from the other species present (Kalscheuer* et al.*, 2010; Syson* et al.*, 2011). All resonances were assigned using authentic compounds. The concentration of each metabolite was expressed as a percentage of dry cell weight.

### *α*-Glucan analyses.

Dry, powdered cells were prepared as described above and 25 mg was re-suspended in 600 µl of 50 mM Tris-HCl, pH 7.4. Cell-free extracts were then prepared as described above. Typically, 300 µl of the cell-free extract was mixed with 580 µl of buffer and 120 µl of Lugol I_2_/KI solution (Sigma-Aldrich) at ambient temperature. After 3 min, absorbance at 500 nm was recorded on a Perkin Elmer Lambda 25 spectrophotometer. The concentration of *α*-glucan was determined with reference to a standard curve with purified *α*-glucan. *α*-Glucan was purified from *S. venezuelae* cells grown on sterile cellophane discs on MYM-TAP plates for 30 h at 30 °C. Harvested cells were boiled in water for 5 min, centrifuged at 4000* g* for 30 min and re-suspended in 10 ml of water. The centrifugation and re-suspension steps were repeated three times. The cells were then lysed by sonication. Cell debris was pelleted by centrifugation at 30 000* g* for 15 min. The resultant supernatant was washed with a 3 : 2 (v/v) mixture of 0.2 M glycine, pH 10.5, and chloroform (5 ml). The aqueous fraction was then washed twice with chloroform. The aqueous fraction was concentrated to ~8 ml using a rotary evaporator and then centrifuged at 108 000***g*** at 4 °C for 4 h. The gelatinous pellet was collected and dissolved in water (5 ml). The *α*-glucan was precipitated with ethanol (1 volume) overnight at 4 °C. The solid collected by centrifugation at 4 000 *g* for 10 min was re-dissolved in water (0.5 ml) and centrifuged at 12 000* g* for 10 min to remove insoluble material. Finally the soluble material was freeze-dried to yield *α*-glucan as an amorphous powder. ^1^H NMR spectroscopy was used to confirm the identity and purity of the α-glucan (Bittencourt* et al.*, 2006; Dinadayala* et al.*, 2004; Ortalo-Magné* et al.*, 1995). A standard curve gave a linear relationship between a known mass of *α*-glucan (up to 1 mg ml^–1^) and the absorbance at 500 nm (up to 0.51) after treatment with Lugol solution as described above. In a separate experiment, cell-free extracts were also analysed using a dot blot probed with a monoclonal antibody raised against the mammalian *α*-glucan glycogen (Baba, 1993). Cell-free extracts were prepared by boiling freeze-dried powdered cells (24 mg) in water (0.5 ml) for 4 h. Cell debris was removed by centrifugation and by passing samples through 0.45 µm filters. Samples (3 µl) were spotted onto nitrocellulose filters before being probed with the antibody (van de Weerd* et al.*, 2015).

### Detection of trehalose synthase activity.

Cells were harvested from MYM-TAP plates after 24 h of growth and cell-free extracts were prepared as described above. A 5 mM solution of maltose in D_2_O was incubated at ambient temperature for 2 h to allow the *α* and *β* anomers to equilibrate and 60 µl was added to 540 µl of each cell-free extract to give a final concentration of 500 µM. The concentrations of maltose, trehalose and glucose were monitored using NMR spectroscopy as described above.

### Transmission electron microscopy.

For the periodic acid-thiocarbohydrazide-silver proteinate (PATAg) staining of *α*-glucan (Thiéry, 1967), single colonies of *S. venezuelae* were cut out of an agar plate and fixed in a solution of 2.5 % (v/v) glutaraldehyde in 0.05 M sodium cacodylate, pH 7.3 (Gordon* et al.*, 1963). Using a Leica EM TP machine (Leica Microsystems), the samples were washed in 0.05 M sodium cacodylate and then post-fixed with 1 % (w/v) OsO_4_ in 0.05 M sodium cacodylate for 60 min at room temperature. After washing and dehydration with ethanol (Beringer* et al.*, 1977), the samples were gradually infiltrated with LR White resin (London Resin Company) according to the manufacturer’s instructions. After polymerization, the resulting material was sectioned with a diamond knife using a Leica EM UC6 ultramicrotome (Leica Microsystems). Ultrathin sections of approximately 90 nm were picked up on 200-mesh gold grids that had been coated in pyroxylin and carbon. The grids were stained for *α*-glucans (Robertson* et al.*, 1975) as follows. They were placed in 1 % (v/v) periodic acid for 20 min at room temperature, washed in water, placed in 0.2 % (w/v) thiocarbohydrazide in 20 % (v/v) acetic acid overnight, washed in acetic acid then water, and finally stained with 1 % (w/v) silver proteinate for 30 min in the dark. After washing in water, the grids were dried and viewed in a Tecnai 20 transmission electron microscope (FEI) at 200 kV and imaged using an AMT XR60B digital camera (Deben).

### Scanning electron microscopy.

Samples of *S. venezuelae* were mounted on an aluminium stub with Tissue Tek optimal cutting temperature compound (Agar Scientific). The sample was then cryopreserved by plunging into liquid N_2_ slush at approximately −210 °C, and transferred to the cryostage of an ALTO 2500 cryotransfer system (Gatan) attached to a Zeiss Supra 55 VP field emission gun scanning electron microscope (Zeiss SMT) or the same type of cryo-system on an FEI Nova NanoSEM 450 (FEI). Surface frost was sublimated at −95 °C for 3 min before sputter coating with platinum for 150 s at 10 mA whilst below −110 °C. Finally, the sample was moved onto the cryostage in the main chamber of the microscope, held at −125 °C, and viewed at 3.0 kV. Digital TIFF files were stored.

### Fluorescence light microscopy.

*S. venezuelae* was grown at the base of glass coverslips penetrating solid MYM-TAP at approximately 45° from vertical for 5 days at 30 °C. The coverslips were left to dry for 10 min in a flow hood and then soaked in 300 µl ice-cold methanol for 1 min, which was rinsed off by dipping in water. An aqueous solution of propidium iodide (25 µg ml^−1^) and wheat germ agglutinin Alexa Fluor 488 conjugate (50 µg ml^−1^; Life Technologies) was applied (25 µl) to the growth line on the coverslip. The samples were then incubated in the dark for 30 min and excess dye was removed by repeated dipping in water for 20 s. The samples were then blotted dry and 9 µl of 20 % (v/v) glycerol was applied to a microscope slide. The coverslip was placed onto the microscope slide and nail polish was applied at the edges of the coverslip to secure it to the microscope slide. The samples were kept in the dark until viewed with a Nikon Eclipse 600 CCD microscope (Cairn) at ×100 magnification with an oil immersion lens. Photographs were taken with an Orca HQ cooled CCD digital camera (Hamamatsu) and digital images were prepared using Image J (NIH) software.

### Stress tolerance analyses.

Wild-type and complemented strains were grown for 4 days and the *glgE* mutant strain was grown for 6 days to maximize the yield of spores and minimize the number of hyphal fragments in each case. Spores were harvested by rolling dry sterilized acid-washed glass balls (50×0.4 mm in diameter) over the lawns of sporulating mycelia. For all stresses apart from desiccation, 15 spore-coated balls were agitated in 1 ml of 50 mM Tris-HCl, pH 7.3, containing 0.001 % Triton X100. The aqueous samples were sonicated for five cycles of 30 s on and 30 s off at 40 % amplitude to disperse the spores in the water and to disrupt any remaining hyphal fragments. The samples (150 µl) were then subjected to various stresses: either lysozyme (0.1 µg) followed by incubation at 37 °C for 30 min, additional sonication with 10 cycles of 30 s on at full amplitude and 30 s off, or heat-shock at 50 °C for 7 min. Samples were serially diluted, spread (100 µl) onto solid MYM-TAP medium and incubated at 30 °C for 1–2 days allowing the number of colony-forming units to be determined. To test for desiccation tolerance, six spore-coated glass balls were agitated in 0.75 ml of water. The aqueous sample was sonicated for four cycles of 15 s on and 45 s off at 40 % amplitude to disperse the spores in the water and disrupt any remaining hyphal fragments. Serially diluted samples (20 µl) were spread on MYM-TAP solid medium and subjected to desiccation stress by incubating them for 8 h in chambers containing anhydrous silica gel giving at atmosphere close to 0 % relative humidity. The severity of each of the stresses used was optimized to give ~75 % spore survival with the wild-type strain to maximize the chances of seeing changes in the mutant strain. Control samples had no stress treatment.

## Results

### GlgE pathway genes are upregulated during sporulation

**Fig. 2.**
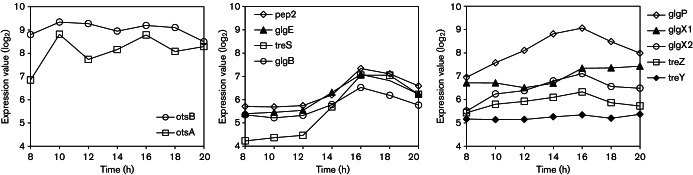
Microarray transcriptional profiling of the genes associated with *α*-glucan metabolism in *S. venezuelae*. The plots show the per-gene normalized transcript abundance (log_2_) as a function of the age of the liquid culture for (a) *otsA* (SVEN_ 4043) and *otsB* (SVEN_ 4041), (b) *treS* (SVEN_ 5096), *pep2* (SVEN_ 5095), *glgE* (SVEN_ 5097) and *glgB* (SVEN_ 5094), and (c) *glgX1* (SVEN_ 5112), *glgX2* (SVEN_ 5898), *treY* (SVEN_ 5896), *treZ* (SVEN_ 5890) and *glgP* (SVEN_ 5104). The developmental stages are indicated, noting that they progress rapidly in liquid culture.

Microarray transcription profiling of *S. venezuelae* throughout development in liquid culture has previously been carried out (Bibb* et al.*, 2012) and the resulting transcriptome data have been deposited in the ArrayExpress database under accession no. E-MEXP-3612. Mining of these data showed that the *otsA* and *otsB* genes associated with the production of trehalose are expressed at a relatively high level throughout development (Fig. 2a). All the genes of the GlgE pathway were clearly upregulated at least twofold as the cells progressed from vegetative growth through to sporulation (Fig. 2b). Finally, most of the genes associated with the recycling of *α*-glucan were at least somewhat upregulated upon sporulation (Fig. 2c).

### Sporulation is delayed in a Δ*glgE* strain

**Fig. 3.**
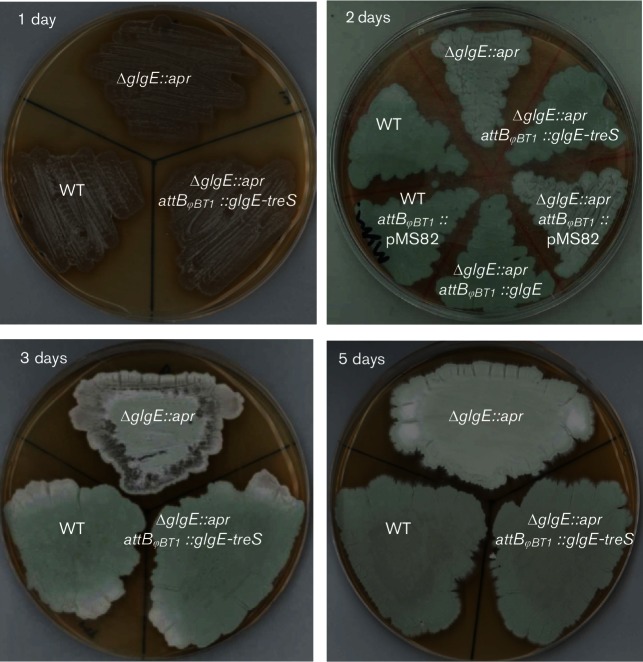
Deletion of *glgE* delays development. Phenotypes are shown of wild-type *S. venezuelae* (WT), WT carrying the empty vector pMS82 (WT *attB_ΦBT1_*::pMS82), the constructed Δ*glgE::apr* null mutant, the mutant carrying the empty vector (Δ*glgE::apr attB_ΦBT1_*::pMS82), the mutant complemented with *glgE* (Δ*glgE::apr attB_ΦBT1_::glgE*), and the mutant complemented with *glgE* and *treS* (Δ*glgE::apr attB_ΦBT1_::glgE-treS*). Note the decreased production of green spore pigment in the null mutant whether carrying empty vector or not. Strains were grown on MYM-TAP solid medium and photographed after 1, 2, 3 and 5 days, as annotated.

To determine the role of GlgE in *α*-glucan metabolism in *S. venezuelae*, we first generated a *glgE* mutant in which the coding region was replaced by an apramycin resistance (*apr*) cassette. The Δ*glgE::apr* mutant formed colonies, but showed decreased production of the green spore pigment (Bush* et al.*, 2013) compared with the wild-type strain, when grown for 2 days on MYM-TAP solid medium with maltose as a carbon source (Fig. 3). Delayed growth was apparent between 1 and 5 days of growth, such that the dry cell weight of the mycelium of the mutant strain was less than two-thirds that of the wild-type strain after 3 days of growth (Fig. S1). Normal growth was restored through complementation with an intact plasmid-born *glgE* gene (Figs 3 and S1), confirming the direct link between the *glgE* gene and the phenotype.

A 4 bp overlap between the ORFs of *glgE* and downstream *treS* genes (Fig. 1b) raised the likelihood of a polar effect on *treS* caused by the replacement of the *glgE* gene by the *apr* cassette. We therefore tested whether the *glgE* mutant possessed any trehalose synthase (TreS) activity by feeding cell-free extracts with maltose and monitoring the production of trehalose using NMR spectroscopy (Fig. S2). Unlike the wild-type strain, it was apparent that the mutant did not produce active trehalose synthase. However, the lack of this enzyme activity appeared to have no impact on the phenotype because normal growth was restored with the reintroduction of the *glgE* gene, whether *treS* was also re-introduced or not (Figs 3 and S1). Furthermore, a *treS* null mutant with an intact *glgE* gene exhibited no growth phenotype (data not shown).

### The Δ*glgE* strain accumulates maltose and *α*-maltose 1-phosphate but no *α*-glucan

**Fig. 4.**
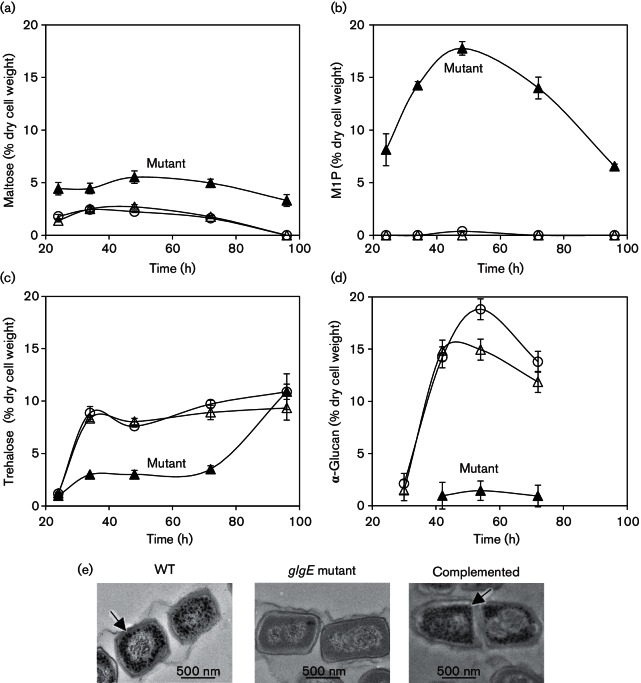
The *glgE* mutant accumulates *α*-maltose 1-phosphate and maltose at the expense of α-glucan and trehalose. The wild-type (WT; open circles), constructed *glgE* null mutant (Δ*glgE::apr*; filled triangles) and complemented (Δ*glgE::apr attB_ΦBT1_::glgE*; open triangles) strains were grown on MYM-TAP solid medium. Cell extracts were analysed by NMR spectroscopy to determine the accumulation of (a) maltose, (b) *α*-maltose 1-phosphate (M1P) and (c) trehalose. (d) The *α*-glucan content was determined using Lugol’s iodine. The data represent means of three biological replicates ± se. (e) Five-day-old colonies were embedded, sectioned and imaged using transmission electron microscopy after staining for *α*-glucan. The arrows indicate stained particles of *α*-glucan.

When grown on MYM-TAP solid medium that contains maltose, the mutant strain accumulated up to double the amount of maltose between 1 and 4 days of growth, compared with the wild-type strain (Fig. 4a). Strikingly, the mutant accumulated ~18 % dry cell weight of *α*-maltose 1-phosphate after 2 days of growth whereas the wild-type accumulated <1 % (Fig. 4b). These observations are consistent with loss of the *glgE* gene and the accumulation of metabolic intermediates immediately upstream of the enzyme GlgE that consumes *α*-maltose 1-phosphate (Fig. 1a). Importantly, the production of this sugar phosphate shows that there were no polar effects on the *pep2* gene coding for maltose kinase. NMR spectroscopy of cell-free extracts of the wild-type strain that had not been boiled to denature enzymes showed that the *α*-maltose 1-phosphate was slowly degraded to maltose (data not shown). This suggests that a phosphatase may make the carbohydrate in *α*-maltose 1-phosphate available in an unphosphorylated state during the later stages of growth.

The mutant accumulated less trehalose than the wild-type strain during the first 3 days of growth (Fig. 4c). This was not due to the lack of trehalose synthase, because it was possible to complement this mutant phenotype with *glgE* alone. These observations indicated that the mutant strain remained capable of generating trehalose through the OtsA–OtsB route. Indeed, after 4 days of growth, the mutant accumulated the same amount of trehalose as the wild-type, when sporulation was underway.

The mutant strain accumulated no or very little α-glucan at any time during growth according to a spectrophotometric method to detect iodine complexes of *α*-glucan polymers (Figs 4d and S3a). This was supported by transmission electron microscopy of samples stained for *α*-glucan (Fig. 4e) and dot blotting with an anti-*α*-glucan monoclonal antibody (Fig. S3b). Importantly, complementation with *glgE* alone restored all the metabolites to their wild-type levels, again consistent with the loss of *treS* expression not having any bearing on the phenotype of this mutant.

### The Δ*glgE* strain produces aberrant spores that are less tolerant of stresses

**Fig. 5.**
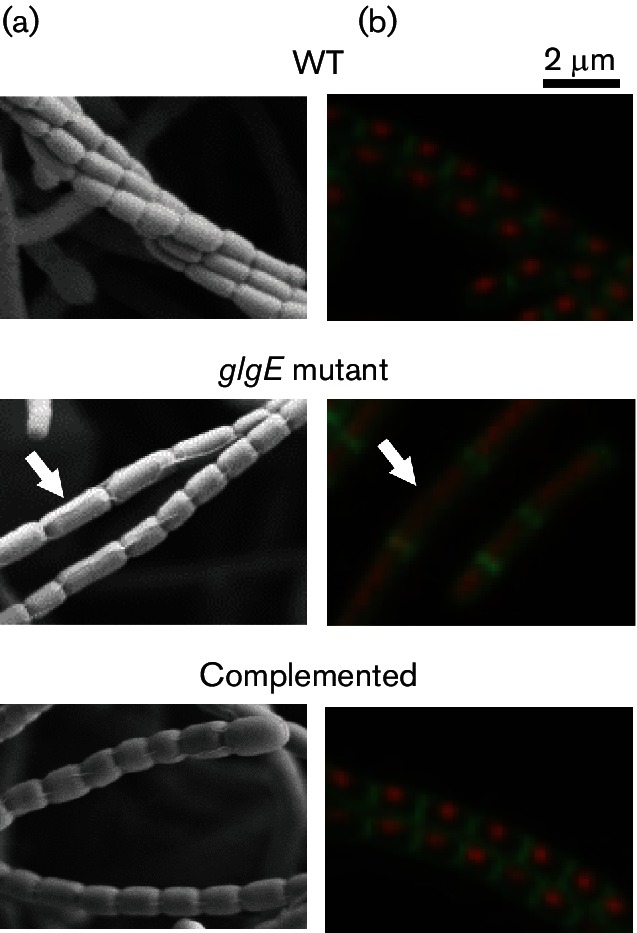
The *glgE* mutant produces aberrant spores of double the normal length and pre-spore cells contain diffuse chromosomal DNA after 5 days of growth. The wild-type (WT), constructed *glgE* null mutant (Δ*glgE::apr*) and complemented (Δ*glgE::apr attB_ΦBT1_::glgE*) strains were grown on MYM-TAP solid medium. Colonies were imaged using either (a) scanning electron microscopy after 7 days of growth or (b) fluorescence microscopy after 5 days of grown with staining for nucleic acid with propidium iodide and cell walls with wheat germ agglutinin Alexa Fluor 488 conjugate. The arrows highlight representative aberrant spores.

Scanning electron microscopy showed that the mutant produced many aberrant spores double the length of normal wild-type spores (Fig. 5a). These accounted for 18±6 % of the spores compared with 2±1 % for the wild-type. Confocal microscopy of cells at the pre-spore stage of development showed that the chromosomal DNA of the mutant was more diffuse than that of the wild-type strain at the same 5 day time point (Fig. 5b). In addition, it appeared as though aberrant spores contained two chromosomes, suggesting the defect was the result of a missing cross-wall. Introduction of *glgE* alone again complemented this phenotype to give a wild-type level of 3±1 % aberrant spores, and condensed DNA in pre-spore cells at the 5 day time point.

**Fig. 6.**
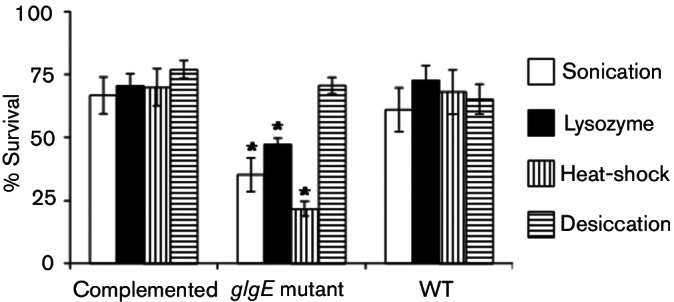
Spores of the *glgE* mutant are less tolerant of some stresses. Spores of the wild-type carrying empty vector (WT, *attB_ΦBT1_*::pMS82), constructed *glgE* null mutant carrying empty vector (Δ*glgE::apr attB_ΦBT1_*::pMS82) and complemented (Δ*glgE::apr attB_ΦBT1_::glgE*) strains were subjected to four stresses: sonication (*n*=6), treatment with lysozyme (*n*=6), 50 °C heat-shock (*n*=6) and desiccation (*n*=3). The survival of spores is expressed as a percentage of control experiments without exposure to stresses. Data from the mutant and complemented strains were compared with those from the wild-type using Student's *t*-test (*P<0.05).

Given the existence of aberrant spores in the mutant strain, we determined if the spores were less tolerant to various stresses. It was clear that they were indeed less able to tolerate sonication, treatment with lysozyme and 50 °C heat-shock (Fig. 6). By contrast, they showed no difference from the wild-type in a regime designed to test desiccation resistance.

### The growth phenotype correlates with the accumulation of *α*-maltose 1-phosphate rather than the lack of *α*-glucan

**Fig. 7.**
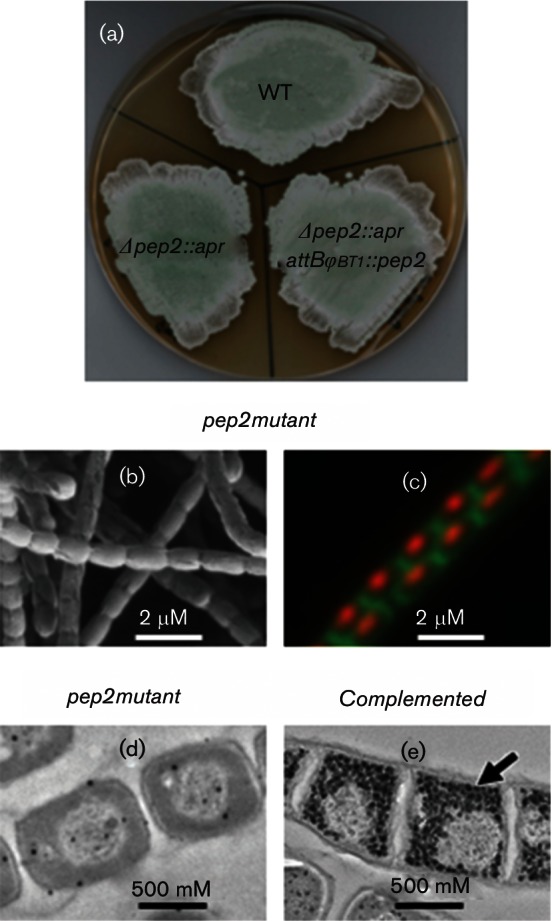
Deletion of *pep2* does not delay growth. (a) Phenotypes are shown of wild-type *S. venezuelae* (WT), the constructed Δ*pep2::apr* null mutant (Δ*pep2::apr*) and the mutant complemented with *pep2* (Δ*pep2::apr attB_ΦBT1_::pep2*). Note that the production of green spore pigment was similar in each case. Strains were grown on MYM-TAP solid medium and photographed after 2 days. (b) A 7-day-old colony was imaged using scanning electron microscopy. (c) A 5-day-old colony was imaged using fluorescence microscopy after staining nucleic acid with propidium iodide and cell walls with wheat germ agglutinin Alexa Fluor 488 conjugate. Five-day-old colonies of (d) wild-type and (e) *pep2* mutant strains were embedded, sectioned and imaged using transmission electron microscopy after staining for *α*-glucan. The arrow indicates stained particles of *α*-glucan.

To establish whether the growth phenotype correlated with the accumulation of *α*-maltose 1-phosphate or the lack of α-glucan, a *pep2* null mutant was constructed. The loss of maltose kinase (Pep2) would be expected to prevent the accumulation of both *α*-maltose 1-phosphate and *α*-glucan. The *pep2* mutant strain did not exhibit a growth phenotype (Fig. 7a), produced normal spores (Fig. 7b) and contained condensed chromosomal DNA in pre-spore cells (Fig. 7c). In these respects, it was indistinguishable from the wild-type strain.

**Fig. 8.**
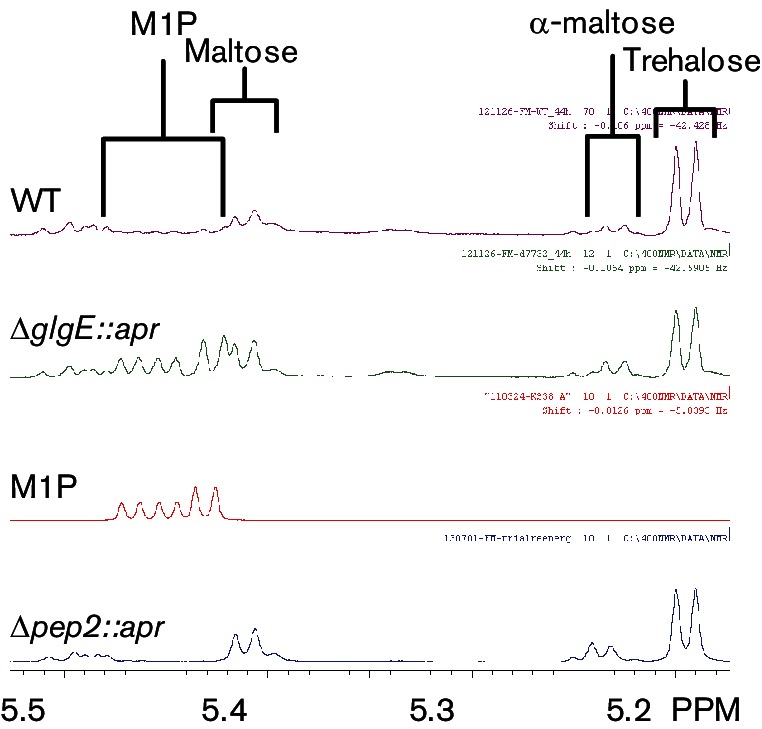
The *pep2* mutant does not accumulate *α*-maltose 1-phosphate. ^1^H NMR spectroscopy shows the accumulation of *α*-maltose 1-phosphate (M1P) occurred in the constructed *glgE* null mutant (Δ*glgE::apr*) but not in the wild-type or constructed *pep2* null mutant (Δ*pep2::apr*) strains. A spectrum of authentic *α*-maltose 1-phosphate (Syson* et al.*, 2011) is also shown for comparison. All three strains also accumulated some of the upstream metabolites trehalose and maltose (both the glycosidic linkage resonance, labelled maltose and the *α*-anomeric reducing end resonance, labelled α-maltose are detected in this region of the spectrum, as described in detail in Methods). Cell extracts were prepared from cells grown on solid MYM-TAP medium for 2 days.

Transmission electron microscopy showed that the *pep2* mutant was devoid of *α*-glucan (Fig. 7d), as predicted (Fig. 1a). A few dark spots were observed with the mutant sample, but these had a smooth edge and were often present in the nucleoids, suggesting these were artefactual rather than revealing the presence of a little *α*-glucan. Indeed, the lack of *α*-glucan in the *pep2* mutant strain was confirmed with dot blotting (Fig. S3b). Complementation with an intact *pep2* gene restored the accumulation of the polymer (Fig. 7e). Importantly, NMR spectroscopy confirmed the lack of accumulation of the product of maltose kinase, *α*-maltose 1-phosphate, in the *pep2* mutant strain, in sharp contrast to the *glgE* mutant (Fig. 8). The trehalose content of the *pep2* mutant was slightly lower than in the wild-type (Figs 8 and S4a). Although this was somewhat reminiscent of the *glgE* mutant, it did not lead to any obvious growth phenotype. The maltose level was only slightly elevated (Figs 8 and S4b) despite the loss of an enzyme that consumes it. Taken these data together, it is clear that the growth phenotype of the *glgE* mutant correlates not with the loss of *α*-glucan but with the accumulation of *α*-maltose 1-phosphate.

**Fig. 9.**
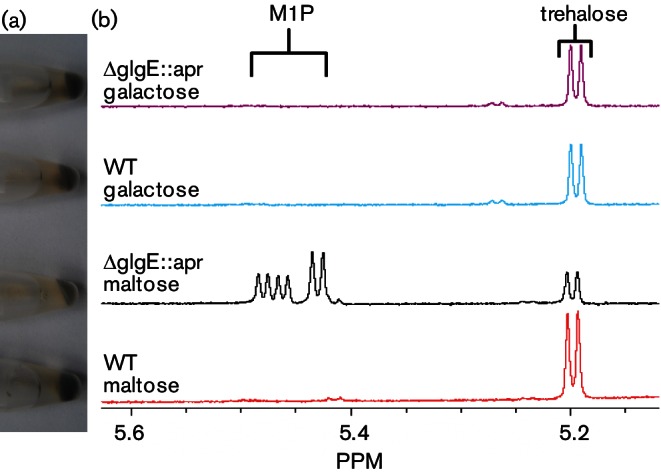
*α*-Maltose 1-phosphate does not accumulate in the *glgE* mutant when grown on galactose. (a) The wild-type (WT) and constructed *glgE* null mutant (Δ*glgE::apr*) strains were grown on solid minimal media containing either galactose or maltose for 9 and 7 days, respectively. The cell pellets were similar in size and colour except for that of the mutant grown on maltose, which was slightly smaller and paler. (b) NMR spectroscopy of cell-free extracts showed that only the *glgE* mutant accumulated *α*-maltose 1-phosphate (M1P), which was at the expense of trehalose.

To corroborate these results, the *glgE* mutant was grown on a carbon source other than maltose with a view to minimizing the accumulation of *α*-maltose 1-phosphate. To this end, the wild-type and *glgE* mutant strains were grown on solid minimal medium containing either galactose or maltose. Growth on galactose was slightly slower with all strains tested but there was no developmental phenotype of the mutant compared with the wild-type strain (Fig. S5). This clearly contrasts with the phenotype observed with maltose. Cells harvested from plates were centrifuged and the size and pigmentation of the pellets reflected the poorer growth on maltose (Fig. 9a). NMR spectroscopy of cell-free extracts showed that *α*-maltose 1-phosphate accumulated only in the *glgE* mutant strain grown on maltose (Fig. 9b). These observations strongly support an association between the accumulation of this metabolite with the growth phenotype of the mutant strain.

## Discussion

It is reasonable to assume that the GlgE pathway is responsible for the formation of *α*-glucan because all four enzymes of the GlgE pathway have been shown *in vitro* to catalyse the appropriate chemistries (Drepper* et al.*, 1996; Garg*et al.*, 2007; Kalscheuer* et al.*, 2010; Miah* et al.*, 2013). In addition, mutation of either the *glgE* or the *glgB* gene in mycobacteria leads to the accumulation of *α*-maltose 1-phosphate (Kalscheuer* et al.*, 2010) and a temperature-sensitive mutation in *Mycobacterium smegmatis* that maps to *glgE* leads to altered glycogen/*α*-glucan metabolism (Belanger & Hatfull, 1999). Furthermore, expression of the GlgE pathway genes increased as *S. venezuelae* progressed from vegetative growth through to sporulation (Fig. 2), consistent with the GlgE pathway being associated with phase II *α*-glucan deposition (Ranade & Vining, 1993). Nevertheless, the production of polymer by the GlgE pathway has not been demonstrated experimentally. Obtaining *in vivo* evidence would be challenging with many bacteria including *M. tuberculosis* and *S. coelicolor* because they possess more than one *α*-glucan metabolic pathway. We chose *S. venezuelae* because it has already been shown to transiently accumulate *α*-glucan (Ranade & Vining, 1993) and possesses only the GlgE pathway genes (Chandra* et al.*, 2011).

The lack of *α*-glucan produced by null mutations in either *glgE* or *pep2* in *S. venezuelae* provides the first demonstration, to our knowledge, that the GlgE pathway is indeed necessary and sufficient for the production of this polymer *in vivo*. In addition, it is clear that *α*-glucan is not essential in this organism, even if growth and development are delayed. Indeed, the ability of the *glgE* mutant strain to produce the trehalose associated with the onset of sporulation (Fig. 4c) shows that it is possible to bypass the normal route to its synthesis via *α*-glucan (Fig. 1a). It seems likely that this is through the recycling of the accumulated *α*-maltose 1-phosphate (Fig. 4b) through dephosphorylation and hydrolysis to allow the liberated glucose to gain access the OtsA–OtsB route.

The developmental delay associated with the *glgE* mutant strain when grown on maltose (Figs 3 and S1) could have been associated with either the lack of *α*-glucan or the accumulation of *α*-maltose 1-phosphate. It was clear that it was the latter because no metabolic or growth phenotype was observed when this mutant was grown on galactose (Figs 9 and S5). In support of this, a *pep2* mutant strain also showed no metabolic or growth phenotype (Figs 7 and 8). It is feasible that the requirement to divert carbon through a less efficient route could be responsible for the growth phenotype. Alternatively, it could be because *α*-maltose 1-phosphate is partially toxic. In support of this, the accumulation of this metabolite in the equivalent mutant strain of *M. tuberculosis* leads to bacterial cell death (Kalscheuer* et al.*, 2010). While bacterial cell death does not normally occur in the *glgE* mutant of *M. smegmatis*, it does when this organism is grown on trehalose because it increases flux through the GlgE pathway. It is clear that the accumulation of *α*-maltose 1-phosphate in *S. venezuelae* is similarly dependent on the carbon source (Fig. 9). However, despite this metabolite accumulating to ~18 % dry cell weight when *S. venezuelae* is grown on maltose (Fig. 4b), it was clearly not to a level that was lethal. Although it is perhaps no coincidence that this intermediate accumulates to the same level as *α*-glucan does in the wild-type strain, it nevertheless attains a remarkably high concentration. If one assumes the dry cell weight comprises 50 % of the wet weight of cells (Bratbak & Dundas, 1984), the concentration of *α*-maltose 1-phosphate exceeds a remarkable 200 mM. Further studies are required to identify the precise reason why *α*-maltose 1-phosphate causes the growth phenotype in *S. venezuelae* and why it is toxic in mycobacteria. The lack of lethality in *S. venezuelae* makes further study more tractable in this organism.

A consequence of the delayed growth phenotype of the *glgE* null mutation was the presence of diffuse chromosomal DNA in pre-spore cells after 5 days of growth (Fig. 5). Given that the chromosome condenses just before spores mature in the wild-type strain, this observation probably reflects the delay in development. More significantly, spores were frequently double the normal length, implying an interruption in the formation of some cross-walls. Such observations have been reported previously whenever the normal process of sporulation has been perturbed (Tzanis* et al.*, 2014). In turn, the spores were less tolerant of various stresses. This may not be due to a lack of trehalose, because the level of this metabolite during sporulation was similar to that of the wild-type strain (Fig. 4c) and the tolerance to desiccation stress was not affected (Fig. 6). The spores were probably compromised in other ways, given the aberrant spore size.

The *α*-glucan produced by the GlgE pathway has a role in the transient storage of carbon/energy during development in streptomycetes (Bruton* et al.*, 1995; Plaskitt & Chater, 1995; Ranade & Vining, 1993; Schneider* et al.*, 2000; Yeo & Chater, 2005). By contrast, *α*-glucans in mycobacteria appear to be associated with either carbon storage at times of nitrogen limitation or immune evasion (Antoine & Tepper, 1969; Cywes* et al.*, 1997; Gagliardi* et al.*, 2007; Geurtsen* et al.*, 2009). Quite what roles *α*-glucans have in other bacteria, particularly those that possess both the classical and the GlgE pathways (Chandra* et al.*, 2011), remains to be seen. Our work with *S. venezuelae* now provides the opportunity to characterize an *α*-glucan derived solely from the GlgE pathway. This will allow the properties of this polymer to be compared and contrasted with that isolated from organisms that possess only the classical glycogen pathway (e.g. *E. coli*) and those with more than one *α*-glucan pathway (e.g. *M. tuberculosis*).

## Supplementary Data

220 Supplementary File 1
