# Supplementary material for: Developmental delay in a Streptomyces venezuelae glgE null mutant is associated with the accumulation of α-maltose 1-phosphate
Source: Microbiology (Reading). 2016 Jul;162(7):1208–19. doi: 10.1099/mic.0.000296 (PMC5042117; doi:10.1099/mic.0.000296)
Supplement: Supplementary file 1 [file mic-162-1208-s001.pdf]

SUPPLEMENTARY MATERIAL

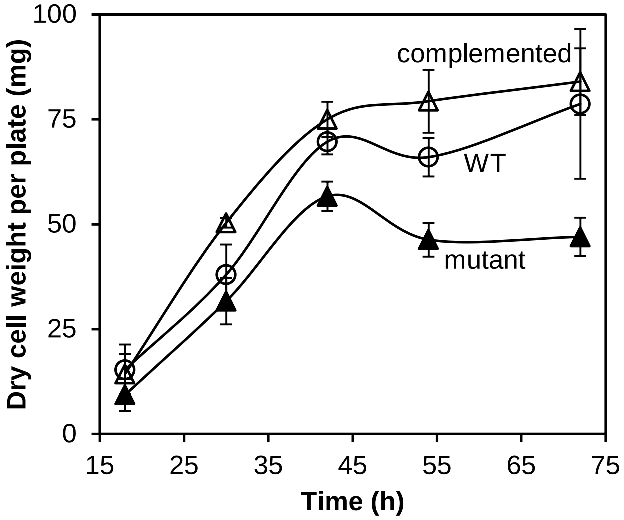

Fig. S1. The *glgE* mutant accumulates less dry cell weight. Plates of MYM-TAP solid medium each covered with a cellophane disc were inoculated with 5×10^6 spores of either the wild-type (WT; open circles), constructed *glgE* null mutant (Δ*glgE::apr*; closed triangles) or complemented (Δ*glgE::apr attB*$_{\Phi BT1}$::*glgE*; open triangles) strains. Cells were scraped off each plate and freeze-dried. The data represent means of three biological replicates ± SE.

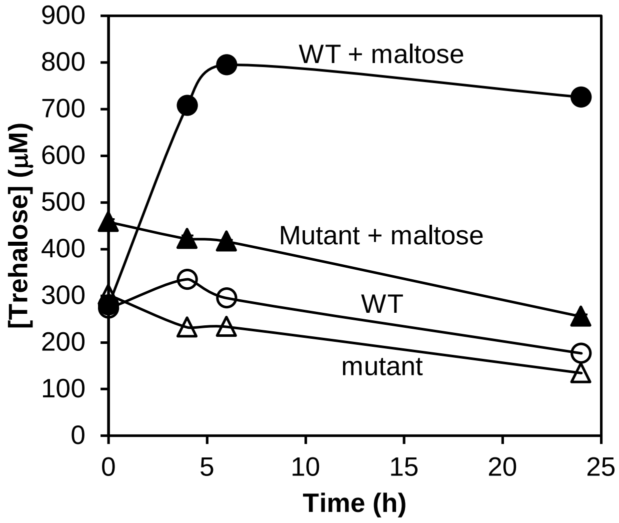

Fig. S2. Trehalose synthase activity is not detected in the *glgE* mutant. Cell extracts were prepared of wild-type (WT; circles) and constructed *glgE* null mutant (Δ*glgE::apr*; triangles) strains that were grown on solid MYM-TAP medium for 2 days. The presence of trehalose synthase was determined by adding maltose (500 µM) and monitoring the concentration of trehalose using NMR spectroscopy (closed symbols). Controls without the addition of maltose (open symbols) were also analysed. Some trehalose was present in the extracts from the start, indicating that the OtsA-OtsB pathway was functional, which is consistent with Fig. 4c. The formation of additional trehalose was only observed with the cell extract from the wild-type strain when maltose was added. The slow reduction in the trehalose concentration over time in each sample could be accounted for by the formation of glucose, implying the presence of a little trehalase activity in each case.

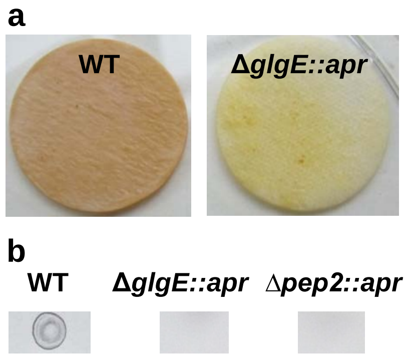

Fig. S3. Deletion of either *glgE* or *pep2* blocks the accumulation of α-glucan. (a) Cell-free extracts of WT and the *glgE* mutant strains grown for 2 days on MYM-TAP solid medium were soaked into glass fibre filter discs and exposed to iodine vapour by placing them in a small glass chamber together with solid iodine. The red-brown colour of the wild-type sample is consistent with the presence of α-glucan. The *glgE* mutant extract gave a significantly paler colour. (b) A dot blot of cell-free extracts of WT and constructed *glgE* (Δ*glgE::apr*) and *pep2* (Δ*pep2::apr*) null mutant strains was probed using a monoclonal antibody raised against the mammalian α-glucan glycogen. Cells were grown on MYM-TAP solid medium for 2 days.

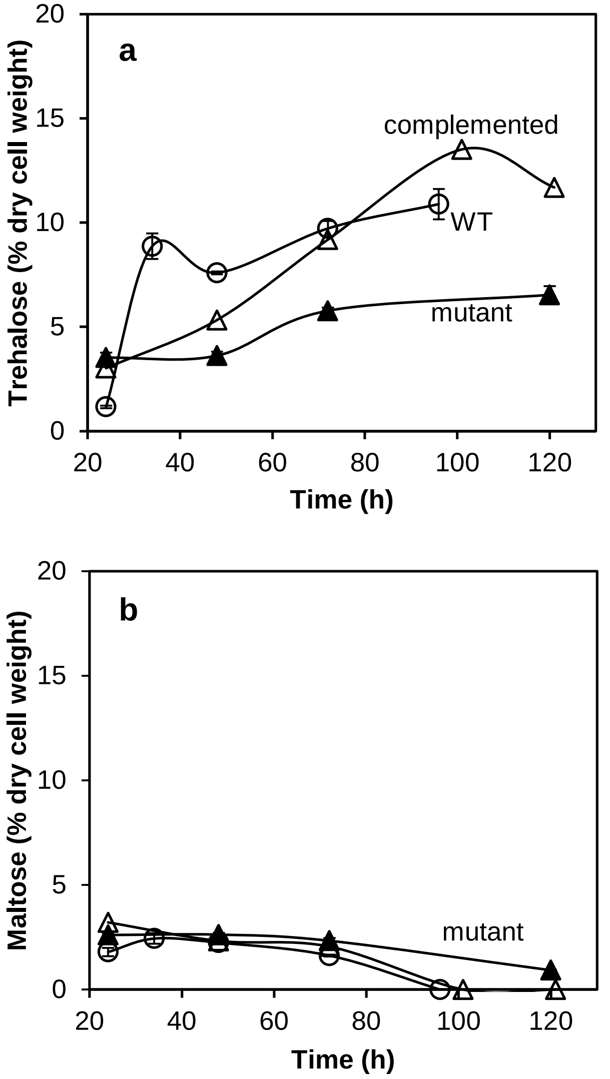

Fig. S4. The *pep2* mutant accumulated less trehalose and a little more maltose. Cell extracts were prepared of the wild-type (WT; open circles), constructed *pep2* null mutant (Δ*pep2::apr*; closed triangles) and complemented (Δ*pep2::apr attB*$_{\Phi BT1}$::*pep2*; open triangles) strains grown on MYM-TAP solid medium. The dry cell weights of (a) trehalose and (b) maltose were determined using NMR spectroscopy. The data represent means of three biological replicates ± SE.

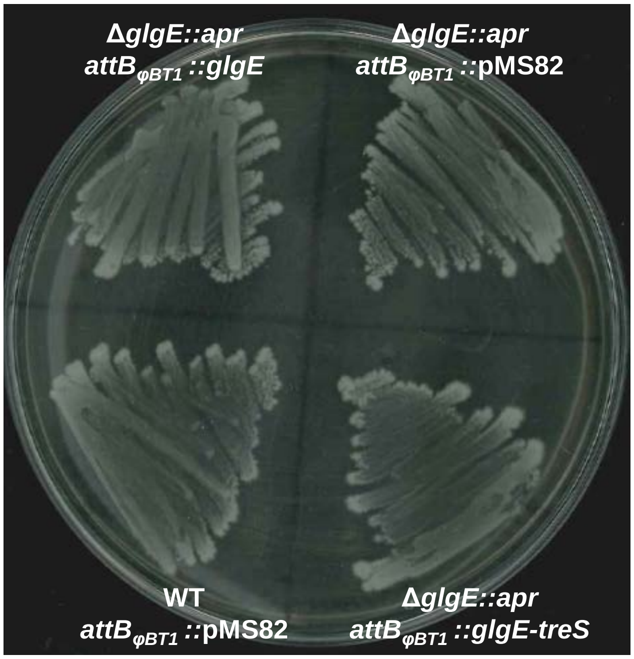

Fig. S5. There is no growth phenotype of the *glgE* mutant strain when grown on galactose. Phenotypes are shown of wild-type *S. venezuelae* carrying the empty vector (WT *attB*$_{\Phi BT1}$::pMS82), the constructed Δ*glgE::apr* null mutant carrying the empty vector (Δ*glgE::apr attB*$_{\Phi BT1}$::pMS82), the mutant complemented with *glgE* (Δ*glgE::apr attB*$_{\Phi BT1}$::*glgE*), and the mutant complemented with *glgE* and *treS* (Δ*glgE::apr attB*$_{\Phi BT1}$::*glgE-treS*). Note that the production of green spore pigment was similar in each case. Strains were grown on minimal solid medium containing galactose and photographed after 9 days.

Table S1. Strains, plasmids and oligonucleotide primers used in this study

Strains	Relevant genotype/comments	Source/reference
Streptomyces venezuelae		
ATCC10712	Wild-type	
FM001	Δ*glgE::apr*	This study
FM001- pMS82 (FM007)	Δ*glgE::apr* with pMS82 integrated at the ΦBT1 attachment site	This study
FM001- pFM1 (FM009)	Δ*glgE::apr* with pFM1 integrated at the ΦBT1 attachment site	This study
FM001- pFM2 (FM008)	Δ*glgE::apr* with pFM2 integrated at the ΦBT1 attachment site	This study
FM002	Δ*pep2::apr*	This study
FM002 - pMS82 (FM010)	Δ*pep2::apr* with pMS82 integrated at the ΦBT1 attachment site	This study
FM002 - pFM3 (FM011)	Δ*pep2::apr* with pFM3 integrated at the ΦBT1 attachment site	This study
FM004	Δ*treS::apr*	This study
Escherichia coli		
ET12567(pUZ8002)	ET12567 containing helper plasmid pUZ8002	Paget *et al.*, 1999
BW25113	Δ(*araD-araB*)*567* Δ *lacZ4787*(::*rrnB-4*) *lacIp-4000*(*lacI*^{Q}), I-*rpoS369*(*Am*) *rph-1,* Δ (*rhaD-rhaB*)*568 hsdR514*	Datsenko & Wanner, 2000
DH5α	F– Φ80*lacZ*ΔM15 Δ(*lacZ*YA-*arg*F)U169 *rec*A1 *end*A1 *hsd*R17 (rK–, mK+) *pho*A *sup*E44 λ– *thi*-1 *gyr*A96 *rel*A1	
**Plasmids**		
pIJ773	Plasmid template for amplification of the *apr oriT* cassette for ‘Redirect’ PCR-targeting	Gust *et al*., 2003
pIJ790	Modified λ RED recombination plasmid [*oriR101*] [*repA101*(ts)] *araBp-gam-be-exo*	Gust *et al*., 2003
pMS82	Plasmid cloning vector for the conjugal transfer of DNA from *E. coli* to *Streptomyces* spp. Integrates site specifically at the ΦBT1 attachment site (Hyg^{R})	Gregory *et al*., 2003
pFM1	pMS82 carrying *glgE* driven from its own promoter	This study
pFM2	pMS82 carrying *glgE* and *treS* driven from the *glgE* promoter	This study
pFM3	pMS82 carrying *pep2* driven from its own promoter	This study
**Primers**	**Sequence 5'-3'**	
glgEdisfor	GACCCGCCATCCGAGTGAACGCGGACAGGAGCGGCCATGATTCCGGGGATCCGTCGACC	
glgEdisrev	TGTCCTCGAAGGTGTCGGGGACGGGCTCGTTGACAGTCATGTAGGCTGGAGCTGCTTC	
pep2disfor	AGTCCATTGCGCACCCCGGGGAAAGGACGCGATGCCATGATTCCGGGGATCCGTCGACC	
pep2disrev	CGGCTGGGCGGAGGAGCCTGGGGCGGGGGGTGGTGGTCATGTAGGCTGGAGCTGCTTC	
treSdisfor	GCGACCGTCCCCGCAGACCGGAGGGTCACCCACATCATGATTCCGGGGATCCGTCGACC	
treSdisrev	CCGTCCCACCCCGGACGTATCCGAACTGACCGGCGGTCATGTAGGCTGGAGCTGCTTC	
glgEconfor	GTCCGGTGTCCGTGCTTGC	
glgEconrev	GTCCCCGTTGCTGTCCTGG	
pep2confor	CATCCGGGACACTCTGCGC	
pep2conrev	GCACCCGAGAAACTCGGCC	
treSconfor	GGAACTCGGCCTCGACTGGC	

treSconrev	GGGTGCGCAATGGACTGTCG
glgEcomfor	CTGGTCGACGGATCCGTCC
glgEcomrev	GTCCCCGTTGCTGTCCTGG
pep2comfor	GGTCAGTTCGGATACGTCCG
pep2comrev	GTCATGGGCTGGTGGAGAGG
treScomrev	GGGACGGGAGGAAACCGTC
